# Accuracy and clinical utility of the compact micro-robot: a retrospective study of 95 cases

**DOI:** 10.3389/fonc.2026.1902506

**Published:** 2026-08-03

**Authors:** Wen Wang, Zairan Wang, Dabiao Zhou, Xiaohui Ren

**Affiliations:** Department of Neurosurgery, Beijing Tiantan Hospital, Capital Medical University, Beijing, China

**Keywords:** brain biopsy, compact, micro-robot, Q300, targeting error

## Abstract

**Background:**

Robotic platforms for stereotactic neurosurgery have evolved rapidly, but compact systems have not been clinically validated in large cohorts.

**Objective:**

To assess targeting accuracy and safety of the compact micro-robot (Q300, 1.5 kg, three-dimensional structured-light registration) across multiple stereotactic procedures, and to contextualize its performance against published data from established platforms.

**Methods:**

We retrospectively analyzed 95 consecutive patients who underwent Q300-guided neurosurgery at Beijing Tiantan Hospital (October 2022–August 2024). Procedures included brain biopsy (n=57), ventriculoperitoneal shunt (n=17), CSF bypass procedures (n=17), and catheter drainage (n=4). The primary outcome was targeting error (TE), defined as the Euclidean distance between planned and actual target points on fused postoperative CT. Entry error (EE) was defined as the Euclidean distance between planned and actual entry points on the skull surface. Secondary outcomes included diagnostic yield, complication rate, and operative time.

**Results:**

The mean EE was 0.92 ± 0.47 mm (median 0.85, IQR 0.55–1.20), and the mean TE was 0.95 ± 0.56 mm (median 0.88, IQR 0.56–1.22). All 57 biopsies yielded conclusive histopathological diagnosis (100% diagnostic tissue acquisition rate). The complication rate was 6.3% (6/95), including 3 hemorrhages managed conservatively and 3 infections after temporal horn bypass. No mortality or permanent neurological deficit occurred. The mean operative time was 73.8 ± 31.1 min (range 17–240) in 95 procedures.

**Conclusion:**

The compact micro-robot achieved submillimetric median accuracy, 100% diagnostic tissue acquisition, and efficient operative times across 95 procedures. Its lightweight design and rapid registration offer practical workflow advantages. The infection rate in temporal horn bypass (3/10) requires further investigation.

## Introduction

Stereotactic accuracy is fundamental to modern neurosurgery. Frame-based stereotaxis remains the reference standard, delivering targeting accuracies of 1.0–2.0 mm across clinical series. However, frame application can prolong preoperative preparation, cause discomfort in awake patients, and limit intraoperative flexibility. In addition, frame-based stereotactic biopsy remains associated with procedural complications; a systematic review reported overall morbidity rates of 3–13% and mortality rates of 0.7–4.0% (1).

Robot-assisted stereotaxy has been pursued as an alternative to conventional frame-based techniques. Contemporary stereotactic robots differ substantially in their physical configuration and mechanical relationship with the patient (2). Large mobile-base systems, including ROSA, Neuromate, Remebot, and SINO, use multi-jointed robotic arms positioned beside the operating table. These platforms provide a broad working range but require dedicated floor space and intraoperative positioning of the robotic arm. In large biopsy series, Liu et al. reported a targeting error (TE) of 1.13 ± 0.30 mm across 700 Remebot assisted procedures (3), and Hu et al. reported a TE of 1.10 ± 0.30 mm in 104 SINO assisted biopsies (4). A meta-analysis of 29 robotic stereoelectroencephalography (SEEG) studies reported a pooled TE of 2.13 mm, although these data reflect multi-electrode SEEG implantation accuracy rather than single-trajectory biopsy accuracy (5). Robot-assisted CSF diversion, including shunt placement, temporal horn bypass and cyst drainage, remains underrepresented in the literature (6).

Miniaturized stereotactic systems position a compact guidance unit close to the patient’s head using a rigid mounting structure, such as iSYS1 (7) and Q300. Their smaller physical footprint may reduce spatial interference around the operating table. The configuration requires rigid cranial fixation and appropriate placement of the head clamp to preserve access to the planned entry point. The published evidence for compact robots remains less extensive than that for established mobile-base platforms (8).

The compact micro-robot (Q300, Sinovation, Beijing, China) weighs only 1.5 kg. Registration uses three-dimensional (3D) structured-light scanning to acquire the patient’s facial surface and match it with the surface reconstructed from preoperative imaging (9).

The robotic arm achieves automated orientation to any planned entry point. The targeting accuracy and safety of the compact micro-robot have not been fully assessed. Patient positioning, brain shift, and intraoperative workflow may all affect *in vivo* performance.

This study aimed to evaluate the accuracy and safety of the compact micro-robot (Q300) in 95 consecutive stereotactic procedures at a single institution. We assessed the targeting accuracy across diverse stereotactic interventions, including brain biopsy, CSF diversion (ventriculoperitoneal shunt, temporal horn bypass, and cyst bypass), and catheter drainage. We further summarized the diagnostic yield for biopsy, complication rates stratified by procedure type, and operative efficiency.

## Materials and methods

### Study design and patient selection

We reviewed consecutive patients who underwent Q300-guided stereotactic neurosurgery at Beijing Tiantan Hospital between October 2022 and August 2024. Inclusion criteria: (1) intracranial lesion or condition requiring stereotactic intervention; (2) procedure performed using the compact micro-robot; (3) complete clinical and imaging data. Exclusion criteria: (1) conversion to a non-robotic procedure during surgery; (2) poor image quality; (3) incomplete data. Data were collected prospectively under an institutional review board approved protocol. Written informed consent was obtained from all patients.

### Robotic system and workflow

**Figure 1:**
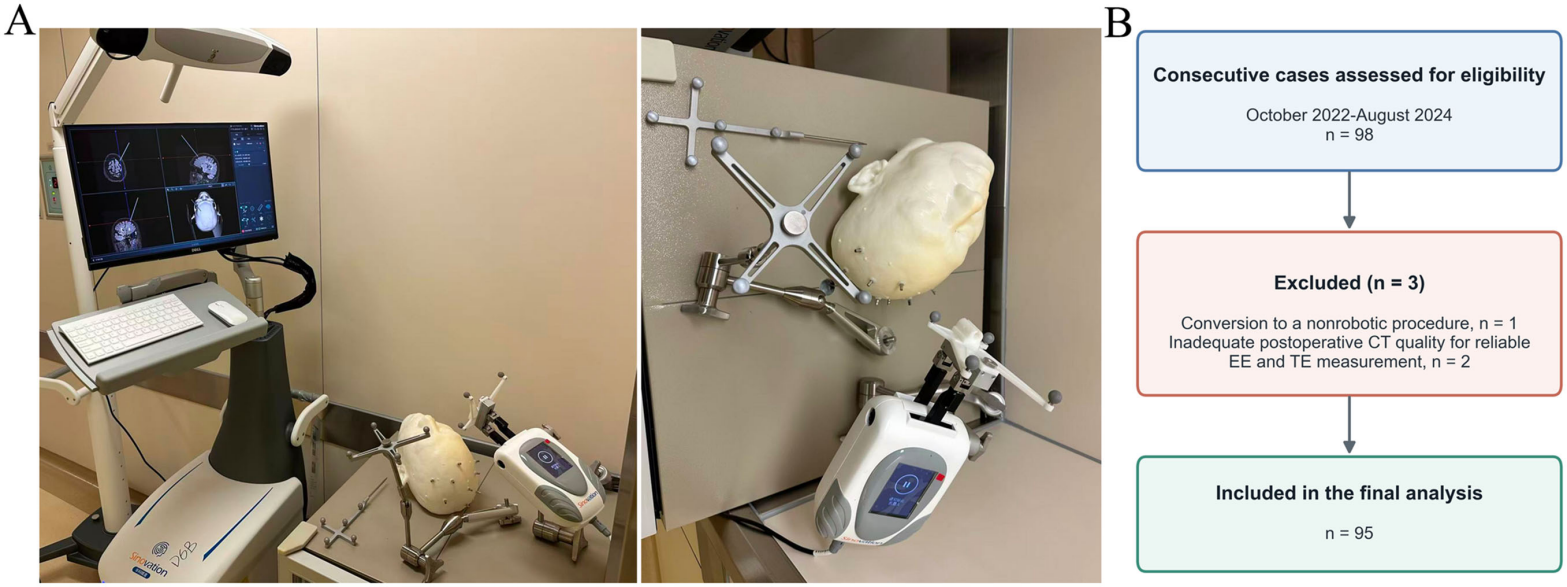
Workflow and patient selection. **(A)** The compact micro-robot system (Q300, Sinovation). **(B)** Patient selection flow diagram.

The micro-robot (Q300, Sinovation, Beijing, China) is a compact robot weighing 1.5 kg. It integrates a structured-light 3D scanner that projects infrared patterns onto the patient’s face, capturing surface contours for markerless registration. The robotic arm achieves automated orientation to the planned entry point (**Figure 1A**).

The surgical workflow was as follows: (1) acquired thin-slice MRI (3D T1, 1.0 mm) and/or CT (1.0 mm) preoperatively; (2) images imported into the navigation workstation (Sinovation), fused, and a trajectory was planned; (3) under general anesthesia, the patient’s head fixed in a Mayfield clamp; (4) 3D structured-light scan performed; (5) registration verified (<1 mm); (6) robotic arm positioned to the planned entry point; (7) surgeon performed the procedure through the robotic guide: a 2.5 mm biopsy needle for biopsy, a ventricular catheter for shunt/bypass, or a drainage catheter for abscess/hematoma.

### Outcome definitions

Postoperative CT (1.0 mm) was obtained within 24 hours with standard brain reconstruction protocol. The postoperative scan was fused with the preoperative plan using the Sinovation navigation workstation. The primary outcome was targeting error (TE), defined as the Euclidean distance between the planned and actual target points at the intended target depth, measured on fused postoperative CT. Entry error (EE) was defined as the Euclidean distance between planned and actual entry points on the skull surface. Two neurosurgeons were blinded to the surgical plan or outcomes, and reviewed all accuracy measurements. The final TE and EE values were recorded after consensus was reached. The secondary outcomes included: (1) diagnostic tissue acquisition rate, defined as successful tissue acquisition sufficient for a conclusive histopathological diagnosis, (2) complication rate, classified using the Clavien-Dindo system, (3) operative time, defined as the interval from skin incision to skin closure.

### Statistical analysis

Data normality was assessed using the Shapiro-Wilk test. EE and TE are reported as mean ± SD, median, and interquartile range (IQR). Ninety-five percent confidence intervals (CI) were computed via bootstrapping (10,000 resamples). Group comparisons used Kruskal-Wallis or Mann-Whitney U tests as appropriate. All analyses were performed using IBM SPSS Statistics software (version 27, IBM Corp.) and *P* < 0.05 was considered statistically significant.

## Results

### Patient characteristics

**Figure 2:**
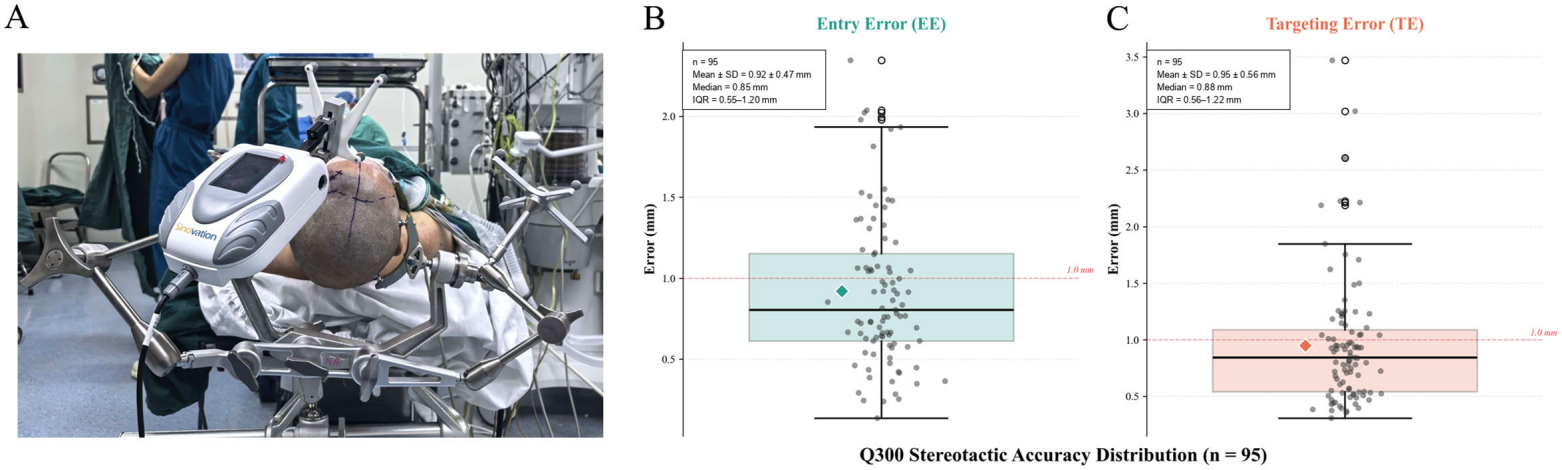
Distribution of entry error (EE) and targeting error (TE) across 95 procedures. **(A)** The intraoperative status of Q300. **(B)** EE. **(C)** TE. The box represents the interquartile range, the horizontal line the median. Individual data points are shown as dots.

A total of 98 consecutive cases were assessed for eligibility. Three cases were excluded. The remaining 95 patients were included (53 males, 42 females; mean age 54.3 ± 14.1 years, range 15–79) in the final analysis (**Figure 1B**). Procedures comprised brain biopsy (n=57, 60.0%; including 2 combined biopsy + Ommaya reservoir placements), ventriculoperitoneal shunt (n=17, 17.9%), temporal horn bypass (n=10, 10.5%), cyst bypass (n=6, 6.3%), abscess drainage (n=3, 3.2%), hematoma drainage (n=1, 1.1%), and frontal horn bypass (n=1, 1.1%). The intraoperative status of Q300 is shown in **Figure 2A**.

Among the 57 biopsies, the final histopathological diagnoses were: glioblastoma IDH-wildtype (n=16, 28.1%), anaplastic astrocytoma WHO grade 4 IDH-mutant (n=5, 8.8%), diffuse astrocytoma WHO grade 2 IDH-mutant (n=2, 3.5%), diffuse midline glioma H3K27M-mutant (n=2, 3.5%), primary CNS lymphoma (n=28, 49.1%), immune-mediated vasculitis (n=2, 3.5%), radiation necrosis (n=1, 1.8%), and demyelination (n=1, 1.8%). Baseline characteristics are summarized in **Table 1**.

**Table 1:** Patient baseline characteristics.

Characteristic	Value
Total patients	95
Sex (M/F), n (%)	53/42 (55.8%/44.2%)
Age (years), mean ± SD (median, range)	54.3 ± 14.1 (57, 15–79)
Operative time (min), mean ± SD (range)	73.8 ± 31.1 (17–240)
Hospital stay (days), mean ± SD (range)	4.5 ± 3.6 (1–30)
Procedure type, n (%)	
— Brain biopsy	57 (60.0%)
— Ventriculoperitoneal shunt	17 (17.9%)
— Temporal horn bypass	10 (10.5%)
— Cyst bypass	6 (6.3%)
— Abscess drainage	3 (3.2%)
— Hematoma drainage	1 (1.1%)
— Frontal horn bypass	1 (1.1%)
Biopsy pathology (n=57), n (%)	
— Glioblastoma IDH-wildtype	16 (28.1%)
— Anaplastic astrocytoma IDH-mutant	5 (8.8%)
— Diffuse astrocytoma grade 2 IDH-mutant	2 (3.5%)
— Diffuse midline glioma H3K27M-mutant	2 (3.5%)
— Primary CNS lymphoma	28 (49.1%)
— Immune-mediated vasculitis	2 (3.5%)
— Radiation necrosis	1 (1.8%)
— Demyelination	1 (1.8%)

### Targeting accuracy

The mean EE of Q300 was 0.92 ± 0.47 mm (median 0.85, IQR 0.55–1.20, 95% CI 0.82–1.02). Mean TE was 0.95 ± 0.56 mm (median 0.88, IQR 0.56–1.22, 95% CI 0.84–1.06) (**Figure 2B**). TE was <1.0 mm in 62.1% (59/95) and <2.0 mm in 94.7% (90/95). All 57 biopsies yielded conclusive histopathological diagnosis.

### Operative time and complications

The mean overall operative time was 73.8 ± 31.1 minutes (range 17–240), and the mean hospital stay was 4.5 ± 3.6 days (range 1–30). For the 38 patients who underwent catheter insertion, the mean operative time was 85.2 ± 36.9 minutes and the mean hospital stay was 4.8 ± 3.2 days. Operative times varied significantly by procedure type (*P* < 0.001): biopsy (including combined procedures), 66.3 ± 24.1 min; ventriculoperitoneal shunt (VPS), 98.5 ± 43.3 min; temporal horn bypass, 89.1 ± 24.2 min; cyst bypass, 61.3 ± 19.9 min; abscess drainage, 46.7 ± 23.1 min; hematoma drainage, 104 min; frontal horn bypass, 60 min (**Table 2**).

**Table 2:** The procedure specific outcomes.

Procedure type	Number	Operative time (min)	Hospital stay (days)	Complications
Brain biopsy	57	66.3 ± 24.1	4.3 ± 3.9	3 (5.3%) hemorrhage
Ventriculoperitoneal shunt	17	98.5 ± 43.3	3.8 ± 1.6	0
Temporal horn bypass	10	89.1 ± 24.2	5.3 ± 4.5	3 (30%) infection
Cyst bypass	6	61.3 ± 19.9	3.5 ± 1.0	0
Abscess drainage	3	46.7 ± 23.1	10.3 ± 3.1	0
Hematoma drainage	1	104	7	0
Frontal horn bypass	1	60	6	0
Total	95	73.8 ± 31.1	4.5 ± 3.6	6 (6.3%)

Operative time defined as skin incision to skin closure.

There were 6 complications (6.3%). Three hemorrhages occurred in the biopsy subgroup (5.3%, 3/57). All were small parenchymal hematomas along the trajectory, managed conservatively without surgical evacuation. Three infections occurred in the temporal horn bypass subgroup (30%, 3/10). All three were surgical site infections requiring antibiotics and one required revision catheter replacement. No mortality or permanent neurological deficit occurred.

## Discussion

In this retrospective study of 95 consecutive Q300-guided stereotactic procedures, we observed a submillimetric median targeting error (0.88 mm), a 100% diagnostic tissue acquisition rate for biopsy, and a 6.3% overall complication rate with no permanent morbidity or mortality. These findings demonstrate that a compact robotic platform could achieve accuracy comparable to that of established systems. To our knowledge, this represents the largest clinical series evaluating a compact stereotactic robot.

**Figure 3:**
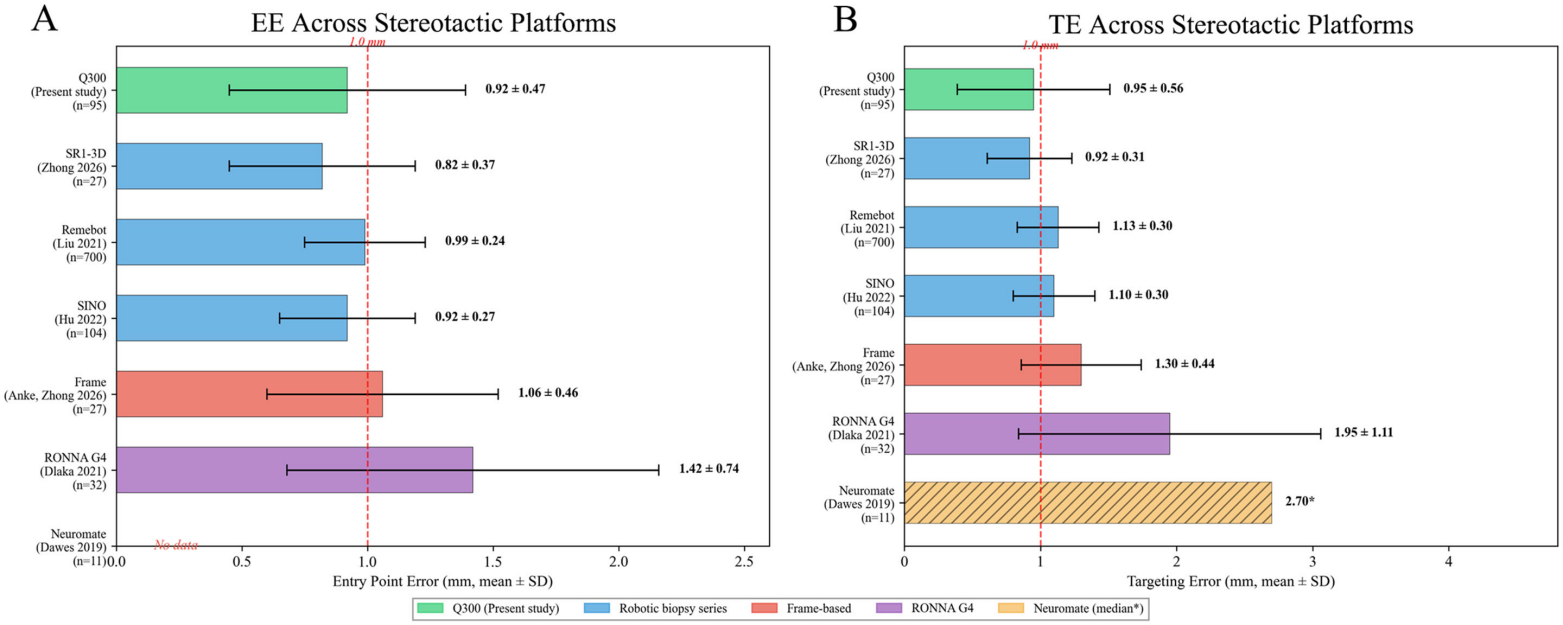
Descriptive accuracy comparison across stereotactic platforms. **(A)** EE. **(B)** TE. Error bars represent ±1 SD where available. The dashed line marks the submillimetric threshold (1.0 mm). Platforms are grouped by procedure type (biopsy series vs. SEEG vs. mixed).

We further compared the accuracy of compact micro-robot (Q300) with published data (**Table 3**). The TE of Q300 (0.95 mm) falls within the range of published biopsy-series data: SR1-3D (10) (0.92 mm), SINO (4)(1.10 mm), and Remebot (3)(1.13 mm). Additionally, the TE of Frame-based (Anke) was 1.30 mm (10). RONNA G4 (11) and Neuromate (12) are labeled accordingly (**Figure 3**). Robotic guidance has been evaluated for hypertensive intracerebral hemorrhage evacuation (13) and brainstem biopsy (14) with Remebot, SEEG implantation with SINO (15), and deep brain stimulation (DBS) lead implantation with ROSA (16). SEEG meta-analysis showed a higher TE (2.13 mm), but these data reflect multi-electrode SEEG rather than single-trajectory biopsy (5). It should be noted that these comparisons are descriptive rather than meta-analytic. Direct statistical comparison is precluded by differences in measurement methodology, patient populations, and procedure types.

**Table 3:** Descriptive accuracy comparison across stereotactic platforms.

Platform	Source	N	Procedure type	EE (mm)	TE (mm)
Q300	Present study	95	Mixed (60% biopsy)	0.92 ± 0.47	0.95 ± 0.56
SR1-3D	Zhong et al., 2026 (10)	27	Biopsy	0.82 ± 0.37	0.92 ± 0.31
Remebot	Liu et al., 2021 (3)	700	Biopsy	0.99 ± 0.24	1.13 ± 0.30
SINO	Hu et al., 2022 (4)	104	Biopsy	0.92 ± 0.27	1.10 ± 0.30
Frame (Anke)	Zhong et al., 2026 (10)	27	Biopsy	1.06 ± 0.46	1.30 ± 0.44
RONNA G4	Dlaka et al., 2021 (11)	32	Biopsy	1.42 ± 0.74	1.95 ± 1.11
Neuromate	Dawes et al., 2019 (12)	11	Biopsy	No data	2.7*

Several design characteristics may be relevant to the observed accuracy of the compact micro-robot. Its 1.5-kg positioning unit is rigidly attached to the Mayfield head clamp, maintaining a fixed mechanical relationship with the patient’s head. 3D structured-light registration (10) provides image-to-patient alignment, and the positioning unit automatically aligns the instrument guide with the planned trajectory. These characteristics may facilitate intraoperative positioning. The mean operative time of 73.8 ± 31.1 minutes provides descriptive workflow data.

Large series have reported shunt revision rates and complication profiles for conventional VPS (17). The 30% infection rate in temporal horn bypass (3/10) is a notable finding. This exceeds typical shunt infection rates of 7.2% per procedure (18), as well as the infection rates in our biopsy subgroup and in comparable robot-assisted biopsy series (19). Possible contributing factors include prolonged catheter dwell time and the small sample size. The finding warrants caution until larger cohorts clarify the risk. Meticulous sterile technique during robot draping and limiting operative time are vital. Larger prospective cohorts are needed to determine whether this infection rate is device-related, procedure-related, or a statistical artifact.

The study has several limitations. The retrospective single-center design limits generalizability. No patient in this cohort underwent Q300-assisted SEEG or DBS. During the study period, SEEG electrode implantation was performed using the SR1-3D system and DBS lead implantation used frame-based stereotaxy at our institution, as these established workflows were already operational. Consequently, the present findings cannot be extrapolated to the feasibility, accuracy, or safety of Q300-assisted SEEG or DBS. The literature comparison is descriptive, not a meta-analysis. It precludes direct statistical comparison due to the differences in measurement protocols, patient populations, and procedure types. Moreover, the catheter insertion subgroup (n=38) is small, and the temporal horn bypass infection estimate is unstable. All procedures were performed by surgeons with prior robotic experience, so the learning curve was not assessed. The clinical status (e.g., GCS, KPS) was not systematically recorded for all patients, limiting functional outcome assessment. The micro-robot platform warrants evaluation in SEEG, DBS, and pediatric populations. Prospective multi-center studies should collect procedure-specific accuracy data, registration error, and standardized complication definitions.

## Conclusion

The compact micro-robot achieved submillimetric median targeting error (0.88 mm), 100% diagnostic tissue acquisition for biopsy, and efficient operative times (mean 73.8 min) across 95 stereotactic procedures. Its accuracy is comparable to that of established robotic platforms. The infection rate in temporal horn bypass (3/10 cases) requires further investigation in larger cohorts. The compact design and markerless registration offer distinct workflow advantages.

## Data Availability

The raw data supporting the conclusions of this article will be made available by the authors, without undue reservation.
